# Telemedicine as the New Outpatient Clinic Gone Digital: Position Paper From the Pandemic Health System REsilience PROGRAM (REPROGRAM) International Consortium (Part 2)

**DOI:** 10.3389/fpubh.2020.00410

**Published:** 2020-09-07

**Authors:** Sonu Bhaskar, Sian Bradley, Vijay Kumar Chattu, Anil Adisesh, Alma Nurtazina, Saltanat Kyrykbayeva, Sateesh Sakhamuri, Sebastian Moguilner, Shawna Pandya, Starr Schroeder, Maciej Banach, Daniel Ray

**Affiliations:** ^1^Pandemic Health System REsilience PROGRAM (REPROGRAM) Consortium, REPROGRAM Telemedicine Sub-committee, Sydney, NSW, Australia; ^2^Department of Neurology, Liverpool Hospital and South Western Sydney Local Health District, Sydney, NSW, Australia; ^3^Neurovascular Imaging Laboratory & NSW Brain Clot Bank, Ingham Institute for Applied Medical Research, Sydney, NSW, Australia; ^4^South Western Sydney Clinical School, The University of New South Wales, UNSW Medicine, Sydney, NSW, Australia; ^5^The University of New South Wales (UNSW) Medicine Sydney, South West Sydney Clinical School, Sydney, NSW, Australia; ^6^Department of Medicine, University of Toronto, Toronto, ON, Canada; ^7^St. Michael's Hospital, Toronto, ON, Canada; ^8^Department of Epidemiology and Biostatistics, Semey Medical University, Semey, Kazakhstan; ^9^Department of Clinical Medical Sciences, The University of the West Indies, St. Augustine, Trinidad and Tobago; ^10^Global Brain Health Institute, Trinity College Dublin, Dublin, Ireland; ^11^Alberta Health Services and Project PoSSUM, University of Alberta, Edmonton, AB, Canada; ^12^Penn Medicine Lancaster General Hospital and Project PoSSUM, Lancaster, PA, United States; ^13^Polish Mother's Memorial Hospital Research Institute (PMMHRI), Łódz, Poland; ^14^Cardiovascular Research Centre, University of Zielona Gora, Zielona Gora, Poland; ^15^Department of Hypertension, Medical University of Lodz, Łódz, Poland; ^16^Farr Institute of Health Informatics, University College London (UCL) & NHS Foundation Trust, Birmingham, United Kingdom

**Keywords:** coronavirus disease 2019, COVID-19, telemedicine, telerehabilitation, telepsychiatry, teleneurology

## Abstract

Technology has acted as a great enabler of patient continuity through remote consultation, ongoing monitoring, and patient education using telephone and videoconferencing in the coronavirus disease 2019 (COVID-19) era. The devastating impact of COVID-19 is bound to prevail beyond its current reign. The vulnerable sections of our community, including the elderly, those from lower socioeconomic backgrounds, those with multiple comorbidities, and immunocompromised patients, endure a relatively higher burden of a pandemic such as COVID-19. The rapid adoption of different technologies across countries, driven by the need to provide continued medical care in the era of social distancing, has catalyzed the penetration of telemedicine. Limiting the exposure of patients, healthcare workers, and systems is critical in controlling the viral spread. Telemedicine offers an opportunity to improve health systems delivery, access, and efficiency. This article critically examines the current telemedicine landscape and challenges in its adoption, toward remote/tele-delivery of care, across various medical specialties. The current consortium provides a roadmap and/or framework, along with recommendations, for telemedicine uptake and implementation in clinical practice during and beyond COVID-19.

## Introduction

Coronavirus disease 2019 (COVID-19) has challenged the status quo of how we approach, deliver, and receive modern medicine (1–4). According to the American Telemedicine Association, telemedicine is defined as “the remote delivery of healthcare services and clinical information using telecommunications technology” (5). It allows for patient care while minimizing the need for physical interaction, thus reducing infection transmission and healthcare facility burden. It can be utilized for ongoing management of chronic conditions, medication compliance, physician-to-patient consultation, and other remote services (3, 4). This can be leveraged to benefit broader populations through telehealth platforms and assisted technologies such as the Internet of things (IoT). Telemedicine and digital technologies demonstrate exceptional potential in improving access and delivery in remote settings. There is also an opportunity to exploit the power of artificial intelligence (AI) algorithms to design a better pandemic preparedness and response plan (6). Health systems have had to adapt to address emerging needs quickly, and many medical subspecialties have transitioned from in-person outpatient care to remote tele- or e-health.

Broadly, telehealth technologies can be deployed for targeted purposes relevant to a pandemic (7). Remote assessment of patients could be undertaken, circumventing visits to outpatient clinics or primary care providers. Patient continuity for those with chronic diseases is essential during a pandemic (3, 4). Such patients are also at high risk of infection and poor outcomes, including mortality, among COVID-19-positive patients (3). Notably, telemedicine also limits infection exposure to healthcare staff, can provide rapid access to subspecialists who are not immediately available in person, and allows for multidisciplinary team discussions. This is crucial in pandemic settings, as the safety of healthcare professionals is essential to ensure the sustainability of health systems to cater to emergent cases and maintain ongoing care. Patients with flu-like symptoms can be triaged, and telemonitoring using video surveillance could be considered for patients who are homebound such as the elderly or frail.

Telemedicine can increase access for certain populations who are challenged during limited healthcare facility visitation, stay-home orders, and quarantine, such as single parents, immunocompromised patients, and patients who rely on the assistance of others for transportation. Monitoring of patients along with remote delivery of home-based exercise, physiotherapy, psychological counseling, social work consultations, and speech and language interventions could be undertaken through telemedicine. Our previous work analyzed the status and deployment of telemedicine during COVID-19 across the geographical divide (Bhaskar et al., under review). In this article, we analyze the uptake of telemedicine across various medical subspecialties and organizational settings with a focus on the current COVID-19 pandemic and propose an operational roadmap for further integration of telemedicine or tele-technologies across health organizations.

## Telemedicine in Emergency Cases and Triage

As hospital systems become strained by the surge of COVID-19 patients, methods to improve the efficiency of emergency departments (EDs) are required, while maintaining standards of patient care. Telemedicine supplies a potential avenue for triage of critical cases. Remote and ambulatory monitoring of patients can allow for remote triage and assessment of emergencies such as acute myocardial infarction (MI), allowing patients to bypass the ED (8). Automated forward triage systems that use algorithms to categorize patients into risk groups could also be utilized, as ED physicians experience considerable time pressure. Current examples include the Multi Sources Healthcare Architecture (MHSA) algorithm and the Electronic Modified Early Warning Scorecard (9). Telemedicine has also been used to triage, expedite, and streamline the local COVID-19 screening process, thereby reducing the strain on healthcare facilities and practitioner exposure.

The New York Presbyterian Hospital, a world leader in digital health innovation, has demonstrated an effective method to reduce the burden of milder presentations (10). They established an ED-based Telehealth Express Care Service, in which after presentation and triage at the ED, patients with milder cases are taken into a private room for a teleconsultation with a physician. Prescriptions and patient instructions are then printed to the room, and the patient is discharged. This dramatically reduces ED waiting times and allows the hospital to deal with ever-increasing ED presentation numbers (10). As patients become anxious about ED infection risk, systems such as these are required, and patients need to be able to effortlessly contact EDs to query whether their symptoms require a presentation.

### Telecardiology

Cardiology is one of the first specialties in which comprehensive telemedicine systems have been implemented. Monitoring of heart rhythm in patients with implanted or real-time wearable devices has allowed ECG with Holter monitoring, echocardiography records, and virtual auscultation. An emerging body of evidence suggesting cardiac involvement in COVID-19 patients has concerned cardiologists (3, 11). This includes cardiovascular complications such as cardiac injury, heart failure, myocarditis, pericarditis, vasculitis, and arrhythmias (12–14). Patients with pre-existing cardiovascular conditions who contract COVID-19 also experience inordinately poor outcomes, including a 5- to 10-fold rise in mortality (15). Due to the COVID-19 pandemic, the American College of Cardiology urgently updated its guidance on “Telehealth: Rapid Implementation for Your Cardiology Clinic,” in which it encouraged remote monitoring and virtual visits of patients with cardiac problems (16). The development of prognostic models based on the recently launched new European register CAPACITY-COVID will help to understand the role of underlying cardiovascular disease (CVD) in patients with COVID-19 (17).

Virtual options can significantly increase efficiency compared to in-person doctor appointments (18). Notably, non-invasive telemonitoring in patients with heart failure reduces all-cause mortality and number of hospitalizations, as well as improves the quality of life (19). In February 2020, the Italian Society of Cardiology published data on the implementation of telemedicine in CVD patients and reported crucial involvement of telemedicine in the prehospital triage for ST-elevated myocardial infarction (STEMI) cases and remote monitoring by primary care physicians (20). An American Heart Association (AHA) statement emphasized the role of telemedicine in pediatric cardiology through advanced video technologies like tele-echocardiography, fetal echocardiography in prenatal diagnosis, screening for congenital heart diseases, and confirmatory echo tests, external rhythm monitoring, catheterization laboratory, and personal tele-electrophysiology (21). Due to their comorbidity risk, efforts to prevent COVID-19 infection in CVD patients should be undertaken seriously by reducing hospital admission and outpatient visits (3).

Treatment adherence is one of the significant issues in the long-term management of CVDs (22). The utilization of mobile phones through mobile health (Mhealth) can be one of the reliable potential solutions in this area through measures such as electronic pillboxes and text reminders (22). The unique advantage of portable devices and smartphones is the ability to reach most patients and caregivers. The widespread use of mobile technologies makes medical support more effective, faster, safer, and less expensive in both outpatient and inpatient settings (23). Mhealth can play an increasingly important role in cardiac care, extensively applied in triage, interventions, management, patient education, and rehabilitation. Telehealth solutions are critical now, as we aim to minimize patients at high and very high cardiovascular risk being hospitalized and provide ongoing support to CVD patients during the COVID-19 pandemic. In Poland, some other systems have been tested in heart failure patients (24, 25), including e-oximeter, allowing for monitoring of heart rhythm and blood saturation, which might help to decide whether those quarantined should be hospitalized during COVID-19.

### Tele–Acute Neurology

Telemedicine allows for prompt assessment of potential emergent neurological cases and can aid those with hospital access issues and those requiring fast acute assessment (2, 4). Acute stroke outcomes are vastly impacted by the speed at which treatment is given, whether it be through tissue plasminogen activator (tPA), endovascular clot retrieval (EVT), or anti-hypertensives. During times of physician shortages, as doctors become re-purposed for COVID-19 purposes, rapid approaches to acute stroke management are needed (2). Reperfusion treatment viability through computed tomography (CT) can be assessed remotely, allowing reperfusion treatment using tPA and/or EVT to be efficiently undertaken. Furthermore, telemedicine can be utilized to determine which patients require an urgent transfer from non-EVT-capable hospitals to EVT-capable hospitals (26). A program developed in Germany known as TRANSIT-stroke, in which rural hospitals established a telemedicine network, saw an improvement in patient outcomes as neurological assessment was made faster, treatments were issued within the required timeframe, and 24 h neurologist access was enabled (27). Similarly, successful programs have been undertaken worldwide, such as telestroke programs in Hawaii and South California (28). There is also evidence to suggest that patients who receive acute stroke assessment through telemedicine do not perceive decreased physician empathy compared to those who receive physical consultation (29). This somewhat relieves concerns about impaired patient–physician connection through telemedicine. While telemedicine decreases the time it takes to analyze head CTs, more work is needed to ensure that this benefit applies equally across different telestroke programs (30).

Mobile stroke units (MSUs) go beyond this to provide CT scanners and stroke personnel within an ambulance vehicle. Such programs exist in locations such as Melbourne (Australia), various states in the US, and Hamburg and Berlin (Germany), among others (31). MSUs improve acute ischemic stroke outcomes by reducing the time to reperfusion; however, further development is needed in the treatment of hemorrhagic stroke. Telemedicine could also allow CT assessment of mild traumatic brain injuries (such as concussions). This can help to determine if the patient requires transfer to a major hospital or can be treated locally and will also allow for post-concussion checkups (32).

## Telemedicine in Critical Care and Respiratory Management With an Emphasis on COVID-19

Vulnerable patients who require respiratory management and/or critical care are at increased COVID-19 risk due to their impaired state and require effective management with the aid of technology (33). In 2019, the Society of Critical Care Medicine (SCCM) Tele-ICU Committee in the United States published an update on developments in telehealth critical care (TCC) (34). They described three emerging trends in TCC: hub-and-spoke structure in which a central hub provides remote technical support, administrative support, and integration to a network of hospitals; decentralized structures in which consultations and patient reviews will be made on a case-by-case and request basis between two sites; and a hybrid structure in which a centralized structure exists but direct contact between spokes can be made for, e.g., specialist consultations. Barriers to TCC included cost and reimbursement issues, lack of responsibility for individual hospitals, and legislative issues (34).

A 2012 systematic review and meta-analysis of telemedicine in the US intensive care unit (ICU) setting demonstrated decreased mortality and length of hospital stay with telemedicine incorporation (35). However, a statistical difference between an active model or high-intensity passive model, in which continuous patient telemonitoring is conducted, and a low-intensity passive model, in which only teleconsultation with an intensivist is conducted, was not ascertained and is an area for further research (35). Patients with respiratory issues are at higher risk of COVID-19 severe infections due to issues such as ventilator reliance and decreased cough function (33). This includes patients with chronic respiratory conditions such as chronic obstructive pulmonary disease (COPD), bronchial asthma, interstitial lung diseases, as well as chronic neurological conditions such as neuromuscular diseases (33, 36). Telemedicine aids respiratory patients through data collection, such as monitoring of vitals and ventilator status, and by transmitting these data for constant monitoring. In the case of under-resourced or under-developed critical care units in low and middle-income countries (LMICs) (Bhaskar et al., under review), frequent international tele-education can serve to upskill doctors and spread critical care knowledge, such as ventilator management (37).

## Chronic Disease, Primary Care, and the Need to Focus on Non-Acute Care

Patients with non-acute diseases require ongoing support and cannot be neglected during COVID-19 times (1, 3, 4, 33). Studies have shown that telemedicine can lead to similar outcomes as face-to-face delivery of care in the management of patients with heart failure, hypertension, and diabetes (38, 39). Ongoing monitoring of these patients is required to prevent acute manifestations, hospitalization, or disease progression (3, 4). The differences within medical subspecialties and individual patients need to be considered, rather than broadly implementing uniform telemedicine approaches across all departments. For example, infectious disease cases can be complicated and require careful consideration of patient history and investigation findings. In these cases, asynchronous consultations, in which the physician reviews data before supplying patient recommendations, will be helpful (40). In other fields such as neurology, cardiology, and endocrinology, real-time, interactive consultations might be more applicable (3, 4).

Patients with neuromuscular issues are particularly at risk due to COVID-19 (4). Patients with motor neuron disease (MND)/amyotrophic lateral sclerosis (ALS) are among those who experience considerable disability and will require multidisciplinary telehealth (4). Types of telehealth include tele-advice, teleconsultation, tele-prescription, videoconferencing, home-based self-monitoring, and remote non-invasive-ventilation (NIV) monitoring. Videoconferencing involves consultation with a health professional, home-based self-monitoring involves taking one's own measurements and submitting them to a physician, and remote NIV monitoring involves remote monitoring of the patient's NIV data (41). The use of telehealth with ALS patients has been shown to be associated with positive benefits such as reasonable adoption rates, personalized data, and efficient consultations (42). Other movement disorders such as Parkinson's disease (PD) also require ongoing multidisciplinary care (43). Established programs such as the Ontario Telemedicine Network, the ParkinsonNet infrastructure in the Netherlands, and that of Kaiser Permanente in the US all display the ability to integrate telehealth into PD patient care (44). Areas for growth include the reimbursement of nursing homes that utilize telemedicine, acceptance by patients and physicians, and reimbursement of at-home telemedicine programs (44). Furthermore, global partnerships can increase international telehealth integration. For example, the International Parkinson and Movement Disorders Society Africa Section, established in the USA, launched a 5-year program to deliver specialist care to disadvantaged areas in Africa using WhatsApp™. Diagnosis of PD could also be aided by telehealth, with the Unified Parkinson's Disease Rating Scale (UPDRS) and Montreal Cognitive Assessment (MoCA) for PD both being able to be performed remotely (45). Such tele-tools have also been recently proposed in the times of COVID-19 for familial hypercholesterolemia patients, who require continuous monitoring of their health due to lifelong high levels of cholesterol and increased CVD risk (46). In migraine and headache patients, telemedicine could be used to assess new headache profiles for possible COVID-19 symptomology or standard outpatient consultations (4, 47).

Cancer patients are another group at risk of COVID-19 infection due to their immunosuppressed states, which could have fatal outcomes subsequent to infection (48–52). Oncologists would use telemedicine for ongoing monitoring and compliance with cancer patients (49, 51). This could be useful in monitoring adverse reactions to ongoing chemo- or radiotherapy, as well as to identify patients who might be at high risk of emergent medical attention, such as those at risk of venous thromboembolism. Cancer patients could also be offered multidisciplinary care, including psychological interventions, physiotherapy, and specialized interventions such as mindfulness training, to improve the overall quality of life (49). Overall, telemedicine offers opportunities for cancer patients to access specialist care in the comfort of their homes. Approaches to the use of telemedicine and mobile technologies in increasing access to novel drugs or interventions through clinical trials should be expeditiously pursued. Telemedicine could also be used in palliative care and end-of-life planning involving patients' carers, family, and multidisciplinary care team (53). Teledermatology is another promising perspective in the diagnosis and monitoring of skin lesions, including cancer (54).

Non-acute ophthalmological telemedicine has been implemented for retinal scans relating to diabetic retinopathy, retinopathy of prematurity, and other non-acute retinal monitoring (55). Fundus scanning and optical coherence tomography imaging are being sent to remote trained healthcare practitioners (HCPs) for evaluation and additionally are being evaluated by AI analysis using deep learning. These non-acute services are also being utilized locally by emergency and urgent care services to a certain extent (55).

Chronic patients must adhere to medications during this time and should not stop treatment regimens without consulting their physician (3, 4). Patients taking immunosuppressants, steroids, or pain medications may be concerned about their COVID-19 risk, and contact with their physicians needs to be ensured. Adherence to medications can be monitored through Mhealth and telehealth means (56). Such examples include digital adherence technologies (DATs) or electronic directly observed therapy (eDOT) for patients with tuberculosis (56). Measures include ingestible sensors, video observation, digital pillboxes, and smartphone applications and have been trialed in China, India, Belarus, and the US (56, 57).

## Identifying Those with Bulbar and Respiratory Weakness

The European Respiratory Society (ERS) task force has described the implementation of remote home mechanical ventilation and physical therapy for patients with chronic respiratory disorders (58). The emphasis is on promoting common standards of clinical criteria as well as analyzing the cost/benefit ratio and evaluating reimbursing rules to implement in different countries (58). Tele diagnosis uses patient data to aid remote diagnosis and can be utilized to identify those with bulbar and respiratory weakness. Telemedicine strategies such as electronic inhalers, chipped nebulizers, self-monitoring through apps, and text reminders increase medicine compliance in patients with asthma, COPD, and cystic fibrosis (CF) (59). Furthermore, the diagnosis of COPD through telemedicine means such as spirometry tracing and teleconsultation provides an opportunity to utilize technology to increase patient care. Further studies are needed to stratify which patients, in terms of severity, will be best suited to a telemedicine management approach. Another area of potential growth is in using AI algorithms to determine developing COPD exacerbations (60). Telemedicine for asthmatics tends to be more focused on treatment compliance and self-monitoring and can be useful in helping patients learn more about their disease, such as recognizing patterns of asthma triggers (61). Other barriers to care include the risk that patient data may be manipulated, networks potentially becoming compromised, and inconclusive data on the benefit of telehealth on specific diseases such as COPD (62).

Obstructive sleep apnea (OSA) is one such disease in which remote monitoring can be utilized to prevent patients from having to spend time in a sleep clinic or respiratory clinic (63). Home polysomnography devices can be used to track patients' breathing and oxygen levels; however, further work is needed to lower the rate of false negatives to the level of in-person sleep clinics (63). A 2018 prospective study of 780 patients used a portable spirometer, with Bluetooth capabilities and connected to a mobile phone application, to trace results and connect the patient to a physician for analysis (64). This allowed the patient's breathing difficulties to be assessed and categorized as asthma, COPD, or normal breathing function (64). This study shows promising results for remote diagnosis of chronic breathing conditions; however, it does not preclude the need for future testing in some more complicated cases. Other smartphone applications have utilized microphones and questionnaires to analyze and detect breathing difficulties associated with other pulmonary conditions such as coughs and lung cancer (65). COVID-19 could impose severe stress on sleep clinics and may limit in-laboratory polysomnography sleep studies for OSA assessments and diagnosis. Home-based tele-polysomnography for OSA assessment could be explored so that the delayed diagnosis and the associated impact on patients could be minimized.

Patients with OSA often require continuous positive airway pressure (CPAP) while sleeping to improve symptoms and achieve proper rest (66). In order to see sustained results, patients need to use CPAP for at least 4 h at night, combined with lifestyle changes such as weight reduction and smoking cessation (66). Low adherence to CPAP remains a continuous problem for OSA patients due to lack of motivation, discomfort, loud noise, and claustrophobia (67). Telehealth provides an opportunity to increase CPAP adherence by monitoring device output data and patient self-tracking of lifestyle factors. When usage falls, the patient can be contacted to discuss their reasons for low adherence and to motivate them to continue use (68).

Telemedicine could be used to monitor bulbar function in patients with a compromised bulbar function such as ALS (4, 69). The rapid decline in bulbar function could be captured using technologies that are useful in delivering specialist multidisciplinary care (69). Other diseases in which bulbar function may be impaired include myasthenia gravis, spinal–bulbar muscular dystrophy, and riboflavin transporter deficiency (4, 70–72).

## Telemedicine-Assisted Follow-up and Rehabilitation

Telemedicine can aid with rehabilitation following acute incidents such as stroke and traumatic brain injury (TBI) (2, 4), as well as chronic conditions that require ongoing rehabilitation efforts such as COPD, CVD, diabetes, and obesity (3). Stroke telerehabilitation programs involving consultations, exercises, games, and therapy aspects have shown positive outcomes such as improving patients' functional abilities and mental health (4). Other benefits include increasing patient motivation and ease due to being in a home setting (73). It is important that patients receive enough support in areas such as technical setup and troubleshooting. The Telerehabilitation in Heart Failure Patients (TELEREH-HF) trial in Poland demonstrated that a 9-week hybrid comprehensive telerehabilitation (HCTR) program consisting of remote monitoring of training at patients' homes was well-tolerated (24, 25). However, the positive effects of the intervention didn't translate into improvement in clinical outcomes over a follow-up period of 12–24 months in comparison to standard care (24).

A 2018 systematic review similarly found that telerehabilitation allowed for equal or more significant patient outcomes than center-based rehabilitation programs in stroke (74). Furthermore, wearable devices can be used in the rehabilitation of various neurological diseases such as stroke, PD, multiple sclerosis, and TBI. Inactivity is associated with various comorbidities and is often a result of chronic neurological disease or acute accident recovery. Remote monitoring through wearable devices can track activity, gait, and any falls throughout rehabilitation (75).

TBI can result in cognitive issues such as sleep disturbance, photophobia, memory, and behavioral changes (76). It is crucial that patients are not discharged without a follow-up plan. A neuropsychological test battery in the few years following moderate-to-severe brain injury and inpatient rehabilitation is vital to assess any cognitive decline and plateau. During COVID-19 times, it is necessary to move outpatient testing of this sort to remote delivery, wherever feasible and while maintaining efficacy. The Brief Test of Adult Cognition (BTACT) has been shown to be effective over the telephone in patients with TBI to assess cognitive state (77). Remote monitoring of physical activity by physiotherapists and patient consultation with neurologists can also be achieved through telemedicine. However, clear guidelines for rehabilitation management and evidence of efficacy through different delivery systems are lacking (78).

Pulmonary rehabilitation is essential for patients with chronic respiratory issues such as COPD and can be achieved through telehealth measures such as monitoring, consultation, and education (79). This is important in COPD, as potential exacerbations need to be monitored, and lower levels of rehabilitation access are associated with increased rates of hospitalization (79). Additionally, personal movement tracking devices involving accelerometers are helpful in tracking patient exercise, which is an essential area of pulmonary rehabilitation (80). Telehealth rehabilitation still faces major hurdles, however, such as cost-effectiveness, patient training, and the lack of regulatory frameworks surrounding personal health devices (80).

## Telemedicine in Palliative Care

According to the WHO, about 40 million people annually need palliative care, and only 14% of them receive it (81). The importance of primary healthcare in palliative care was highlighted by the first WHO global resolution on palliative care in 2014. The Project ECHO (Extension for Community Healthcare Outcomes), as one of the examples, shows the potential of telemedicine in the training of patients, their family members, and medical workers in palliative care (82, 83). The training of palliative care via telemedicine/telehealth for outpatients in primary care will increase the coverage and quality of both care and life for these patients. Telehealth, including mobile applications, plays a role in making patients more adherent to both pharmacological and non-pharmacological therapies; in remote monitoring of clinical parameters such as cardiovascular and respiratory system function; as well as in monitoring of diet and physical activity. Given the overload of respiratory diseases and the flu-like presentations in routine practice, telemedicine offers an alternative that is particularly relevant in the COVID-19 era.

## Telemedicine/Telehealth Application in Mental Health

Mental health support to frontline health workers, patients, and carers will be crucial, as long isolation, lack of social interaction, as well as anxiety over one's own and others' health will take a toll on well-being (2–4, 84). Psychotherapy, psychiatry, and counseling are easily converted to a teleconference format through platforms (such as—but not limited to—Zoom™ and Skype™) and should be utilized by frontline health workers, patients, and carers where necessary (85). Anecdotal evidence also suggests that patients experiencing paranoid, anxiety, or post-traumatic stress disorders, who may be particularly affected by the COVID-19 climate (84), may feel more comfortable undergoing telepsychiatry over in-person psychiatry. Online delivery will further help to resolve issues such as lack of access to practitioners in rural settings and cultural and linguistic barriers (86). Furthermore, psychoeducation and mental well-being advice can be leveraged through smartphone apps and digital outreach programs (87). These services will become increasingly crucial in the pandemic setting, as physical isolation and frontline work pose both access issues and mental health stressors. The ethics of such teleservices needs to be ensured, with patient confidentiality, referral and billing practices, and physician eligibility being upheld (88). Psychiatrists, psychotherapists, and psychologists need to ensure that they are maintaining their own mental health during this time, with programs such as professional supervision being of help (4).

## Telehealth/Telemedicine for the Elderly

In 2018, nearly one-fifth of the European population was aged over 65 years old (89). An aging population has put significant pressure on public spending; therefore, telemedicine can improve the scale and efficiency of delivery and ongoing management of elderly patients. Elderly patients with mild cognitive impairment or dementia who might be at high risk of an acute condition should be identified using mobile technologies and telemedicine, and telemedicine solutions for the elderly should be easy to use and possibly automatic (4). This would avoid unnecessary burdens to public health facilities. Telemedicine can also be used to act as an interface of the local nursing care staff, carers, and patients with medical specialists. Elderly patients will benefit from remote allied health delivery. Patients who have had a recent surgery could be monitored at home or in nursing care facilities, preventing extended hospital stays. Elderly patients with diagnosed mental health conditions could also benefit from telemedicine. However, self-efficacy and digital literacy presumably have a significant impact on the uptake of telehealth among the elderly (90).

Recent data from the US confirm that the most vulnerable age group for COVID-19 is people over 65 years old, and the highest mortality is observed in those aged 85 and older (91). In Ontario, Canada (as well as in Italy and the US), 54% of deaths related to COVID-19 occurred in retirement homes and long-term care (92, 93). Strict zero-visitation policies have had debilitating effects for some elderly patients, particularly those with dementia (4). Telemedicine has been utilized to connect family members with these patients to prevent further decline in mental status and provide comfort. This is useful, as family members have voiced concerns that physically distanced visits such as through windows may further confuse their loved ones.

Telehealth allows continual monitoring of vitals, physical examination, ongoing clinical management, and communication with patients. In elderly patients with limited accessibility, telemedicine could provide an alternative, easy-to-access service. Elderly patients often suffer from social isolation, and telehealth can bring a sense of community. Furthermore, by using AI, falls can be detected among elderly patients (94). AI can provide personalized medicine solutions to help identify patients at risk of harm. Primary healthcare physicians and nursing homes should watch for signs of depression in the elderly, particularly as it has been shown that telemedicine is competent in managing depressive symptoms in the elderly (95).

## Telehealth/Telemedicine for Congregate Settings

Telemedicine can be useful in delivering interventions in congregate settings (96, 97). Challenges in congregate settings include high population density, limited mobility, built environment issues, and limited access to health. This can make the prevention and management of COVID-19 onerous while preserving human rights and ethical issues. Some of the potential target populations include refugees and migrants (96), those living in incarceration, orphanages, old-age homes, or childcare centers; and schools. These populations are especially vulnerable to infection such as COVID-19, where an outbreak can have facility-wide implications and adverse health consequences and fatality. A simulation study on the possible impact of COVID-19 outbreak in a Bangladeshi refugee camp found a dire need for dramatic increases in healthcare capacity and infrastructure (97). Existing approaches to control an outbreak, should it occur, would not be practically feasible, necessitating innovative solutions as well as novel and untested strategies in humanitarian settings (97). Telepsychiatry to monitor and deliver interventions in congregate settings, especially among refugee populations living in resource-constrained areas (98, 99), could be an alternative when traditional therapy is not possible. Telepsychiatry programs for congregate settings should be developed, and further studies are needed to evaluate their long-term impact on patient monitoring and care (99, 100).

## Cost–Benefit Analysis

Telemedicine systems are not novel concepts and have been used to good effect for programs such as forward triage in EDs, critical care monitoring, and physician communication. Existing systems will need to be reallocated, and innovations will be pushed through in order to provide care across all medical fields and to reduce hospital burden. This needs to be achieved within the constraints of funding, legislation, and supply-chain barriers. Temporary government funding will be necessary to roll out telemedicine to both rural and urban settings, as well as relaxations to legislation that allow practitioner reimbursement of telemedicine services (101). A study by Sayani et al., addressing the cost and time barriers in chronic disease management through telemedicine in LMICs, found telemedicine to be economically beneficial not only by reducing the socioeconomic barriers to cost and access but also by increasing the uptake of services (102). Another systematic review of studies conducted on costs of home-based telemedicine programs from 2000 to 2017 found that home telemedicine programs reduced care costs, although detailed cost data were either incomplete or not presented in detail (103). The data on the cost-effectiveness of telemedicine solutions in different medical areas remains inconsistent and confounded by many variables, including the type of disease and “digital maturity” of healthcare systems. However, in critical situations such as the COVID-19 pandemic, telemedicine is proven necessary, and costing, billing, and reimbursement solutions are needed.

## Reimbursement of All Telehealth Providers

There are variations in reimbursement policies across regions and healthcare systems. One of the major barriers has been harmonizing a standard reimbursement policy that is acceptable to all stakeholders and sustainable. We recommend that an integrated framework involving public and private parties could help develop a less complicated and streamlined reimbursement structure. Notably, the adoption of a “flip the switch” health insurance strategy in North Carolina to reimburse telehealth visits “at parity” with conventional office visits for all healthcare providers and specialists is timely and essential. In the long term, the impact of these strategies on healthcare quality and healthcare costs needs further study. Healthcare providers must lead the way here in the COVID-19 crisis to explore innovative approaches such as B2B monitoring.

## Limitations of Telemedicine

Certain limitations may act as roadblocks in the uptake, implementation, and scale-up of telemedicine and supporting technologies. Considerable training is required to ensure patients can familiarize themselves with video teleconsultations and the use of supportive technologies. Physicians also need targeted technical, clinical, and communication training based on their subspecialty needs. Issues of limited access to broadband and Internet facilities are an area that particularly limits the deployment of telemedicine in remote areas and under-resourced settings. Telehealth requires reliable broadband access, which is not always acceptable both for clinics in rural areas and for patients living in such areas. When using telemedicine technology, legal restrictions and a lack of clarity as to what is permitted are possible, and these restrictions force telemedicine providers to proceed with caution. Some conditions are not considered in the legislation of health systems. It is still not entirely clear whether virtual consultations and video surveillance will be fully paid in hospitals or will be evaluated as shorter visits so that the rates will be lowered. Physician licensing and stability of the telemedicine infrastructures are issues of relevance in under-resourced settings.

Several critical medical procedures cannot be replaced by telemedicine, nor can it be offered to everyone, and there are many excluded groups of patients, including those with deficiencies (e.g., deaf and blind patients) and elderly patients. The effectiveness of telemedicine relies on the possibilities of the implementation of these tools in the given hospital/healthcare system, preparations/training of physicians/nurses, and awareness of the patients.

Beyond this, when introducing technologies and measures to overcome gaps in the healthcare system, it is essential not to simply ask, “where are the gaps,” but also to define the standards and ideals of care and continually iterate toward these ideals. As mentioned before, telemedical consultations do not approach the same level of fidelity that an in-person physical exam yields, between physical exams, body language, vocal intonations, and odors. As such, the fidelity of the technology involved with telemedical consults must continually iterate to reach the same level of fidelity and information that an in-person visit might yield. In this vein, virtual and augmented reality technologies, while evolving, hold promise for the future of telemedicine, particularly in envisioning a future in which high-fidelity physician and patient “avatars” may meet in a virtual space for a telemedical consult, replicating aspects of an in-person visit through immersive technologies.

## Conclusion and Future Discussion

**Figure 1:**
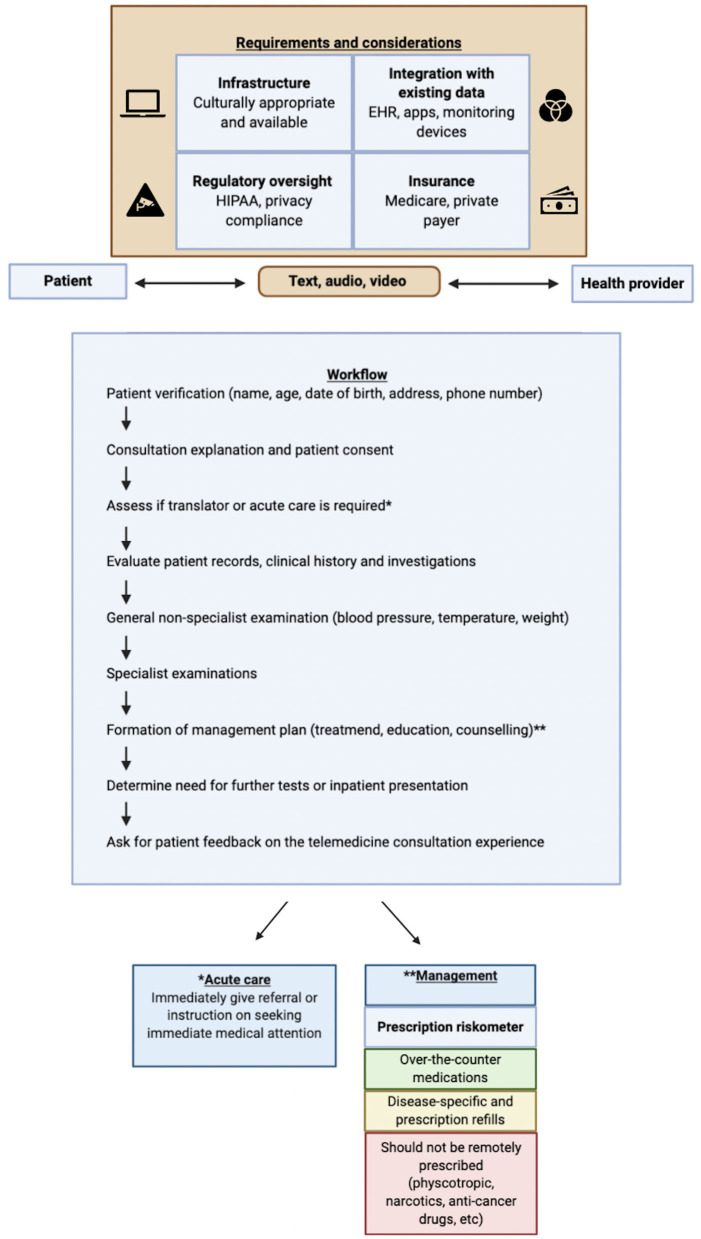
Various requirements and considerations for streamlined telemedicine implementation and the Pandemic Health System REsilience PROGRAM (REPROGRAM) consortium workflow for routine teleconsultation and management of patients. Patients and healthcare providers can interact through telemedicine via text, audio, or video means. Effective telemedicine has several requirements, including culturally appropriate and available infrastructure; regulatory oversight and privacy compliance such as through the Health Insurance Portability and Accountability Act of 1996 (HIPAA); integration of technologies with existing data such as electronic health records (EHRs), apps, and monitoring devices; and insurance coverage such as Medicare or private-payer schemes. Credentialing on both sides is essential. The consultation should start with verification of the patient's identity through name, age, phone number, date of birth, and address. The physician should then clearly specify that this is a telemedicine consult and that no audio or video of the communication will be recorded. It is imperative that health record information is protected. The physician should then clearly and explicitly ask for consent, whether that be verbal, text, or video. At the start of the consultation, the physician should assess if acute care is required and make a cursory determination if telemedicine consultation is sufficient. If necessary, the physician should supply an immediate referral or advise the patient to seek immediate medical attention. During a typical consultation, the patient will be evaluated; and specific diagnostics and treatment would be recommended based on the assessment of the healthcare provider; and follow-up could be scheduled either in person or virtually. The physician should go through records, clinical history, and investigations including pathology and diagnostic reports, and obtain any additional information that the patient can provide. A general, non-specialist examination should be obtained, and any vital signs that the patient has the means to measure should be gathered.

COVID-19 has expedited the uptake of telemedicine across various specialties. The rapid move by various bodies, associations, and providers to use telemedicine in maintaining patient continuity while limiting COVID-19 risks of exposure to patients and healthcare workers will have a long-term impact well-beyond the current pandemic. Teleconsultation needs are varied across specialties, and therefore, specialty-specific guidelines and recommendations need to be developed. A scoping list of various telemedicine studies across medical subspecialties (telemedicine vs. standard care) has been provided in Table 1. A comprehensive workflow that critically profiles various telemedicine enablers has been proposed in Figure 1, and recommendations to improve various factors are listed in Table 2.

**Table 1:** Telemedicine across various medical subspecialties.

	**Telemedicine studies**	**Outcome parameter**
Telemedicine in emergency cases and triage	Brennan et al. (104)	Reduced average patient throughput time (from admission to discharge).
	Dharmar et al. (105)	Improved physician-rated quality of care.
Telecardiology	Molinari et al. (106)	Reduced hospitalizations in patients with suspected life-threatening cardiac events.
	Khader et al. (107)	Improvement in the quality of life 2 months after the first visit.
	Scalvini et al. (108)	Possible cost reduction due to increased appropriateness of hospital admission and of diagnostic testing.
	Sable et al. (109)	Positive impact on referral patterns and time management in pediatric cardiology practice without increasing the utilization of echocardiography.
Teleneurology	Capozzo et al. (110)	Feasible to triage amyotrophic lateral sclerosis (ALS) patients using telemedicine. Increase in practice outreach and efficiency, especially in COVID-19 times.
	Ohta et al. (111)	Significant improvement in the state and trait anxiety inventories (STAI) scores in Parkinson's disease (PD), ALS, and spinocerebellar degeneration (SCD) + multiple system atrophy (MSA) patients.
Tele–acute neurology	Medeiros de Bustos et al. (112)	Reduction in secondary interhospital transfers.
	Vatankhah et al. (113)	Immediate impact on clinical decisions.
	Lyerly et al. (114)	Telemedicine increased access to acute stroke care.
	Dharmasaroja et al. (115)	Telemedicine increased intravenous thrombolysis rates without compromising favorable and safety outcomes.
	Schwab et al. (116)	Comparable mortality rates and functional outcomes for telemedicine-linked community hospitals and stroke centers to the results from randomized trials.
Telemedicine in critical care and respiratory management	Yang et al. (117)	Telemedicine feasible to support acute management of children who present to community hospitals.
	Kuipers et al. (118)	Acceptable positive predictive value of the electronic inhalation monitoring devices (EIMDs) in patients with respiratory diseases.
Chronic disease and primary care	Orozco-Beltran et al. (119)	Telemonitoring program effective in reducing high risk for rehospitalization or an emergency department visit.
	Doñate-Martínez et al. (120)	Telemedicine program improved self-reported quality of life and decreased use of health resources in elderly patients with chronic diseases. High satisfaction levels also observed in patients on the program.
	Martín-Lesende et al. (121)	Telemonitoring of in-home patients with heart failure and/or chronic lung disease led to an increase in the percentage of patients with no hospital admissions. Trend toward reduced total and cause-specific hospitalizations and hospital stay.
	Palmieri et al. (122)	No significant impact of home-based telemonitoring program on all-cause hospitalization/mortality. Telemonitoring associated with higher patient compliance and achievement of therapeutic targets.
Identifying those with bulbar and respiratory weakness	Pinto et al. (123)	Home-based telemonitoring of non-invasive ventilation decreased healthcare utilization in patients with ALS.
	Paganoni et al. (124)	Video visits associated with marked adjusted cost savings for patients and institutions.
Telemedicine-assisted follow-up and rehabilitation	Rawstorn et al. (125)	Telehealth exercise-based cardiac rehabilitation is as effective as center-based rehabilitation for improving modifiable cardiovascular risk factors and functional capacity.*Note: Systematic review included 11 clinical trials*.
Telemedicine in palliative care	Kuntz et al. (126)	Electronic family (e-family) meetings to facilitate in-patient palliative care during coronavirus disease 2019 (COVID-19) pandemic feasible and well received by families.
	Nemecek et al. (127)	Telemedicine augmented palliative care feasible in patients with advanced care and their family carers. Significant reduction in anxiety levels in the telemedicine group vs. the standard care.
Telemedicine in mental health	Ruskin et al. (128)	Telepsychiatry and in-person treatment of depression have comparable outcomes. Equivalent levels of patient adherence, patient satisfaction, and healthcare cost.
	Salisbury et al. (129)	Telehealth service leveraging non-clinically-trained health advisers supporting patients in use of Internet resources was both acceptable and effective compared with usual care.
	O'Reilly et al. (128)	Clinical outcomes of telepsychiatry equivalent to standard of care.
	Chipps et al. (99)	Telepsychiatry programs are feasible in congregate settings.

**Table 2:** Various factors in telemedicine implementation and corresponding recommendations from the consortium.

**Factors**	**Recommendations**
Physician licensing	Facilitation and harmonization of inter-state licensing. Specialty-specific considerations for telemedicine-based care.
Bandwidth and infrastructure	Mobile phone–based Internet to avail of telemedicine, mobile Wi-Fi routers. The concerted effort through a public–private partnership involving key corporate players to improve Internet penetration in these pockets. Need domain experts to ensure that technologies are appropriately deployed.
Reimbursement, cost, and availability	Medicare-for-all coverage. The government needs to provide funding to develop platforms with minimal expenditure.
Clinician uptake	Specialty-specific training for clinicians and user-centric technology design considerations for easy and improved access.
Physician leadership	Quality control, regular re-skilling of professionals involved, ensuring infrastructure is being monitored. Collaboration between various stakeholders, including providers and insurance payers.
Language barriers	Technologies such as mobile apps that can be easily incorporated into telemedicine workflow.
Privacy	Blockchain-based platforms or applications could safeguard the privacy of physicians.
User training and technology deployment/access to hardware	Appropriate training addressing bias and nuances toward adopting telemedicine. Funding support and rapid deployment of telemedicine technologies across all providers. Provision for delivery of “bundled” telemedicine services. Public and private partnerships can act as enablers. Performance evaluation, ongoing assessment of patient and provider experiences, and adapting to address gaps.
Liability	Developing clear-cut guidelines to mitigate communication and technology-specific risks and liability. This should be done in consultation with providers, patients, and insurance/payers.
Geographical limitations	Use of low-bandwidth applications. Change management and culture-specific support.
Under-resourced settings	Technology-based health promotion, leveraging mobile health applications for community outreach, healthcare buddies who liaise with the community to educate and inform about technology and use. The WHO needs to take the lead in ensuring penetration of telemedicine in under-resourced locations in collaboration with philanthropic partners.
Complex cases	Evidence-based guidelines and workflow recommendations. Various boards, associations, and bodies should formalize standard protocols that could clearly delineate *do*s and *don't*s of clinical examination, diagnosis, and management using telemedicine technologies.
Patient–doctor relationship	Communication tool kit or handouts to improve user experience. Privacy and communication to patients that clinical data and consultations would remain confidential is critical.

The proposed workflow (Figure 1) provides a practical telemedicine framework cognizant of relevant requirements and considerations, and a step-by-step pathway to streamlined telemedicine delivery. This could be used as a template (for further customization or adaptation) by individual medical subspecialties. Current challenges and recommendations to improve telemedicine include (130): (i) infrastructure capacity [formation and expansion of dedicated telemedicine units and workforce; cloud-based infrastructure to support telemedicine associated bandwidth traffic; liability, maintenance, and safety of telemedicine platforms; ongoing and regular maintenance and servicing of telemedicine hardware and software; awareness, education, and training to build confidence about telemedicine use among providers and consumers; compulsory telemedicine modules for medical students and continued professional development (CPD) workshops/courses for healthcare providers and medical informaticians/technologists; targeted courses aimed at re-skilling clinicians]; (ii) integration with existing data (standardized patient-specific information and consent form with telemedicine opt-in/out option); (iii) regulatory oversight issues (setup of telemedicine regulatory authority; accreditation/licensing of providers using telemedicine; guidelines for telemedicine use in inter-state and -nation settings; standardization of telemedicine related technologies and services with regulatory oversight, audit, and reporting; appropriate measures and oversight to protect privacy, security, and confidentiality of patient data; legal frameworks for telemedicine-specific information storage, sharing, and access); and (iv) insurance/payers (guidelines for telemedicine insurance; streamlined payment facilities for making and receiving payments; bundled services payments and insurance coverage).

Another important and emerging area is the use of text messaging [short message service (SMS) or multimedia message service (MMS)] as a model for service delivery (131–136). Text messaging has proven efficacious in diabetes self-management, smoking cessation, weight loss, physical activity, and adherence to medication regimens [such as in human immunodeficiency virus infection and acquired immune deficiency syndrome (HIV/AIDS) patients who are on antiretroviral therapy] (132). A systematic review on text messaging interventions identified the following issues: identification of intervention characteristics, ensuring intervention effects last over a longer duration of time, and cost-effectiveness of these interventions (132). Issues of privacy and security are also poignant in this context. Nevertheless, text messaging offers potential benefit as a public health intervention toward chronic disease management (133–136), medication adherence, and secondary prevention (134).

Perceptions and experiences/satisfaction, regarding telemedicine services, of the patients and providers is important in improving telemedicine implementation, delivery, and impact (137–141). A systematic review on patient satisfaction with telemedicine highlighted methodological deficiencies in published studies (137). A study on patient and clinician experience with telemedicine found that virtual video visits may provide effective follow-up and increased convenience in comparison to routine in-person visits (139). Another study found a perception of patients with type 2 diabetes that telemedicine can improve their access to care (140). Further studies focusing on communication issues and the quality of interpersonal relationships during telemedicine consultations and how these factors affect healthcare delivery using this medium are required (137, 141).

Some specialist examinations, including neurologist consultation, can also be conducted. The American Academy of Neurology has issued guidelines for telemedicine consultation (142). Physicians can assess mental status; any visual, auditory, or cognitive deficits; comprehensive speech; cranial nerves; apparent tremors; and gait. Motor examinations can also be conducted with the aid of a caregiver in order to help ascertain strength, tone, reflexes, dermatome sensation, and cerebellar function. In such a case, consent must be gained from both the patient and the assistor. Special considerations may apply for pediatric patients or adults with intellectual disabilities. Based on the severity of symptoms, the patient may require a management plan, including specific treatment, health education, and counseling if necessary. Patients can be prescribed ongoing prescriptions, specific medications, or add-on medication to optimize regimes, given that there is no ambiguity about diagnosis and the medications are not dangerous. If there is any ambiguity about diagnosis, this must be recognized as a limitation of this mode of telemedicine, and documentation must be made. Further tests should be done or referred for in-person consultation if necessary. It should be noted that detailed examination of tone, strength, and reflexes; comprehensive eye examinations; and examinations that require specific maneuvers such as vestibular examinations should be avoided, as examination findings won't be accurate. These recommendations will also need to be adjusted according to individual state or federal legislation. The future of telemedicine beyond the current COVID-19 pandemic will depend on how we address existing challenges, building resilient health systems (2–4). Further randomized controlled trials to evaluate the long-term effects of telemedicine-based interventions in various patient populations should be planned. Telemedicine will play a major role as a “safety net” during the pandemic.

## Author's Note

The COVID-19 pandemic is causing an unprecedented public health crisis impacting healthcare systems, healthcare workers, and communities. The COVID-19 Pandemic Health System **RE**silience **PROGRAM** (**REPROGRAM**) consortium is formed to champion the safety of healthcare workers, policy development, and advocacy for global pandemic preparedness and action.

## Author Contributions

SBh devised the project, the main conceptual ideas, including the proposal for a new telemedicine workflow, the proof outline, and coordinated the writing and editing of the manuscript. SBh and SBr wrote the first draft of the manuscript. SBh encouraged SBr to investigate and supervised the findings of this work. All authors discussed the results and recommendations and contributed to the final manuscript.

## Conflict of Interest

SP is the Vice President of Immersive Medicine at Luxsonic technologies, a medical technology company specializing in virtual/augmented reality for medical education, collaboration, and training. The opinions expressed in this article are those of the authors and do not necessarily represent the decisions, official policy, or opinions of the affiliated institutions. The remaining authors declare that the research was conducted in the absence of any commercial or financial relationships that could be construed as a potential conflict of interest.
